# Giant small bowel lipoma presenting as concurrent volvulus and intussusception: a rare cause of acute abdomen

**DOI:** 10.1093/jscr/rjag779

**Published:** 2026-09-05

**Authors:** Washington Javier Cisneros-Caicedo, Erika Deyanira Montenegro-Garcia, David Ignacio Narváez-Salas, William Vicente Portilla-Yepez, Juan Eduardo Ortiz-Carrasco, Gabriel Alejandro Molina-Proaño, Xavier Raul Mantilla-Pinto, Karen Lisseth Checa-Yugsi, Tatiana Paola Campoverde-Salamea, Bryan David Velasco-Ruiz

**Affiliations:** Universidad Internacional del Ecuador, Av. Jorge Fernández S&N y Av. Simón Bolivar, Quito 170411, Ecuador; Department of General Surgery, Hospital Padre Carollo, Av. Rumichaca S33-10, Quito 170702, Ecuador; Department of General Surgery, Hospital Padre Carollo, Av. Rumichaca S33-10, Quito 170702, Ecuador; Pontificia Universidad Católica del Ecuador, Av. 12 de Octubre y Vicente Ramón Roca, Quito 17051, Ecuador; Department of General Surgery, Hospital Vozandes Quito, Av. Juan José de Villalengua Oe2-37, Quito 170521, Ecuador; Department of Pathology, Hospital Padre Carollo, Av. Rumichaca S33-10, Quito 170702, Ecuador; Department of Diagnostic Imaging, Hospital Padre Carollo, Av. Rumichaca S33-10, Quito 170702, Ecuador; Universidad San Francisco de Quito (USFQ), Diego de Robles y av. Pampite, Quito 170901, Ecuador; Postgraduate Program in Anesthesiology, Pontificia Universidad Catolica del Ecuador, Av. 12 de Octubre 1076 y Vicente Ramón Roca, Quito 170511, Ecuador; Department of General Surgery, Hospital Padre Carollo, Av. Rumichaca S33-10, Quito 170702, Ecuador; Pontificia Universidad Católica del Ecuador, Av. 12 de Octubre y Vicente Ramón Roca, Quito 17051, Ecuador; Department of General Surgery, Hospital Padre Carollo, Av. Rumichaca S33-10, Quito 170702, Ecuador; Pontificia Universidad Católica del Ecuador, Av. 12 de Octubre y Vicente Ramón Roca, Quito 17051, Ecuador; Department of General Surgery, Hospital Padre Carollo, Av. Rumichaca S33-10, Quito 170702, Ecuador; Pontificia Universidad Católica del Ecuador, Av. 12 de Octubre y Vicente Ramón Roca, Quito 17051, Ecuador

**Keywords:** giant lipoma, intestinal obstruction, volvulus, intussusception, small bowel

## Abstract

Gastrointestinal lipomas are rare benign tumors whose clinical relevance increases when they exceed 4 cm, raising the risk of mechanical obstruction or vascular compromise. Highlighting this atypical presentation is crucial: early recognition enables prompt segmental resection, preventing critical vascular compromise while definitively excluding synchronous malignancy. The report of this case is essential to optimize the approach to atypical presentations and ensure timely surgical treatment. In this study, we present a case of a giant lipoma in the jejunum which caused intussusception and volvulus of the small bowel.

## Introduction

Gastrointestinal lipomas (GL) are benign tumors of mesenchymal origin, primarily composed of mature adipose tissue. Although most are incidental findings, their clinical importance lies in the complications they can generate, such as intussusception (II), obstruction, and volvulus. Their reported incidence in studies ranges between 0.035% and 4.4% [1]. The anatomical distribution of GL shows a marked predilection for the large bowel, where 65% to 75% of cases are located, followed in frequency by the small bowel (SB) at 15%–20%.

In the SB, prevalence shows an upward progression toward the ileocecal valve, concentrating 50%–60% in the distal ileum, 25%–30% in the jejunum, and 10%–15% in the duodenum [2, 3].

The clinical relevance of these tumors arises when they reach dimensions exceeding 4 cm, also known as giant gastrointestinal lipomas (GGL). With the presence of these lipomas, 75% of patients develop mechanical obstructions, and II accounts for 1%–5% of obstruction causes in adults. Secondary small bowel volvulus represents an extremely rare and diagnostically challenging complication. This entity differs from simple II by presenting early and fulminant vascular compromise, progressing rapidly toward ischemia and segmental necrosis if emergency surgical intervention is not performed [4, 5].

We present a case of a GGL in the jejunum, their complications, and how it was surgically resolved.

## Case report

### Case 1

A 48-year-old female patient, with a history of a single abdominal surgery (a cesarean section), presented to the emergency department with diffuse abdominal pain (VAS 9/10), nausea, and frequent vomiting, with a clinical course of 48 hours of evolution. The patient mentioned experiencing pain with similar characteristics for the past 6 months. She denied constitutional symptoms. On physical examination, the abdomen was distended, diffusely tender to palpation, and exhibited signs of peritoneal irritation.

**Figure 1:**
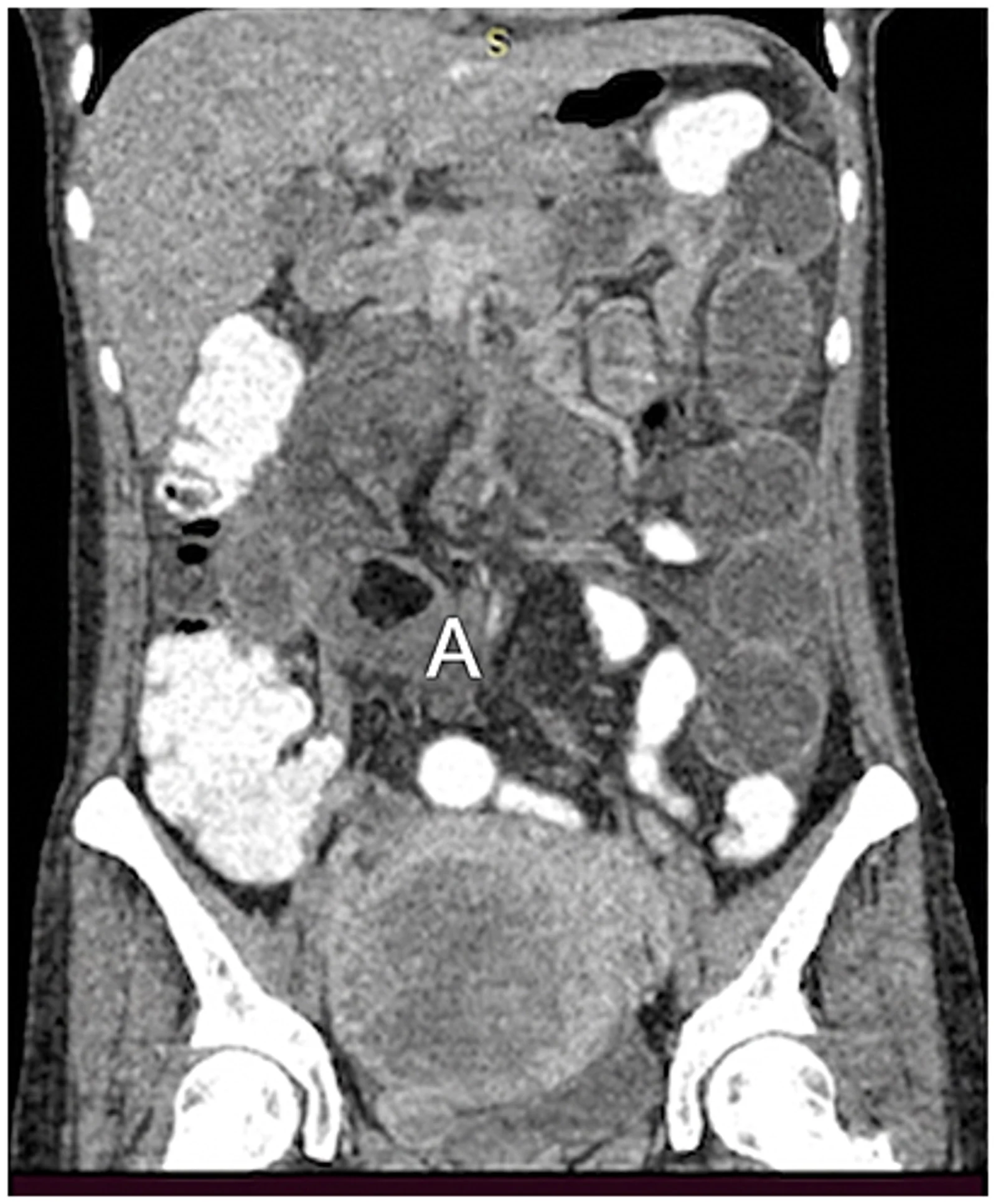
CECT of the abdomen and pelvis (coronal view): Mural thickening and an exophytic intraluminal mass with fat attenuation are observed in the distal ileum, suggesting a submucosal lipoma as the primary diagnostic probability, indicated by ‘A’.

**Figure 2:**
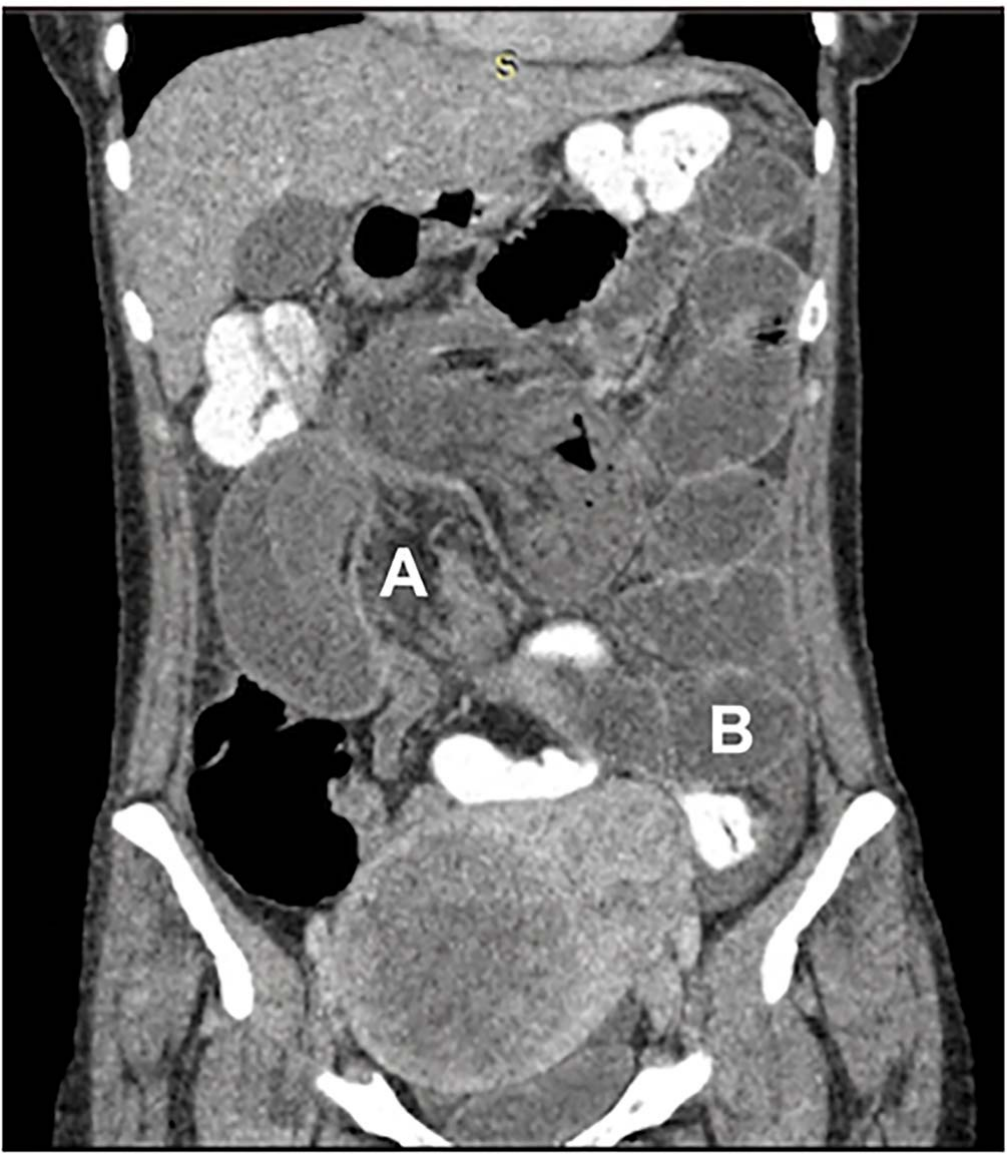
CECT of the abdomen and pelvis: Intussusception of jejunal loops with wall edema is shown at ‘A’. A concurrent large uterine myomatosis is noted at ‘B’.

A non-contrast and contrast-enhanced computed tomography (CECT) scan of the abdomen and pelvis revealed mural thickening of the SB, along with an exophytic intraluminal mass suggestive of a submucosal lipoma in the distal jejunum (Fig. 1), and findings compatible with jejunal intussusception (double-track sign) (Fig. 2).

**Figure 3:**
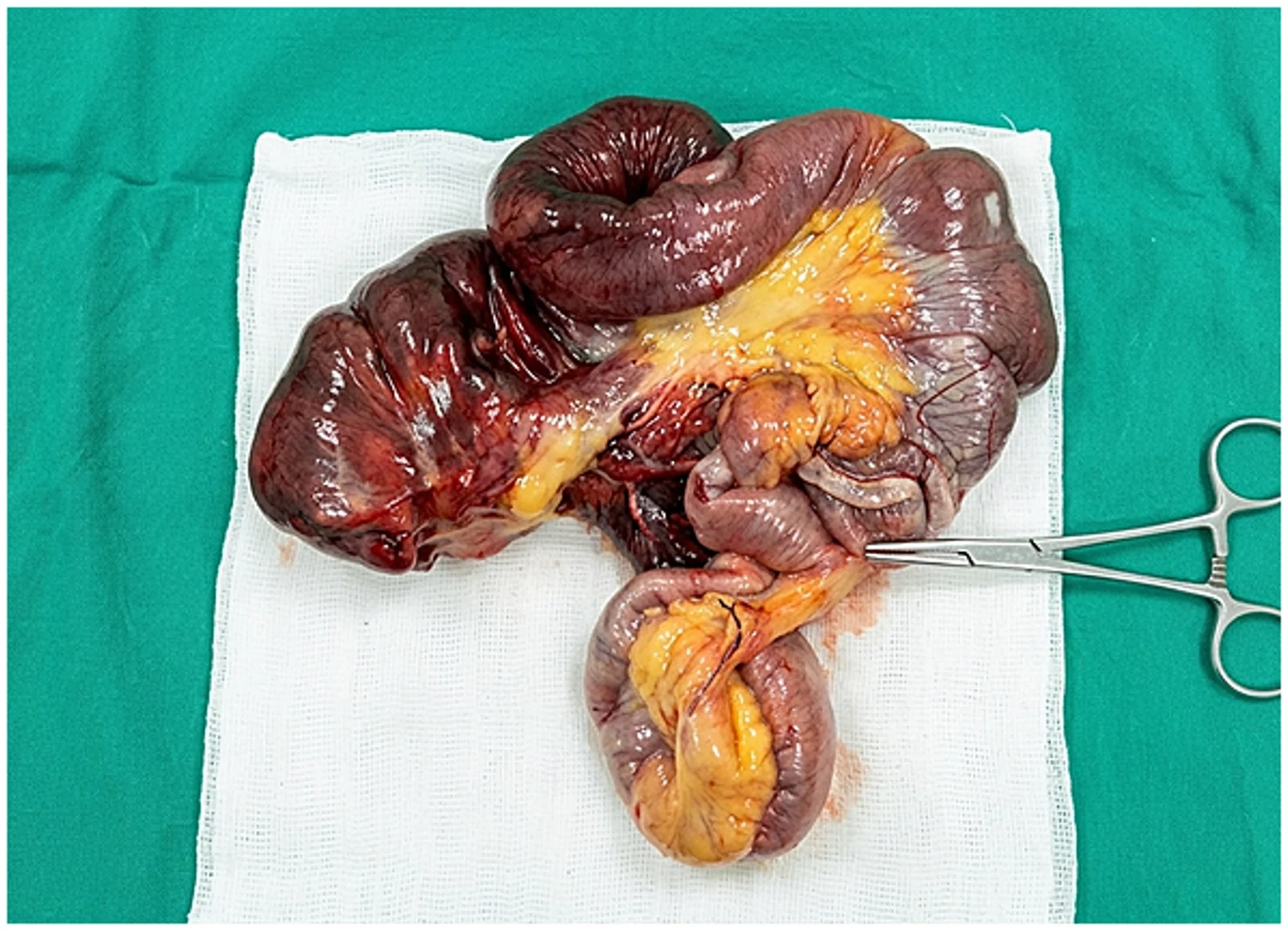
Resected gross specimen: Necrotic segment of the small intestine resulting from a 270-degree jejunal volvulus.

A diagnostic laparoscopy was planned, which revealed II associated with SB volvulus and dilated intestinal loops; this presentation prevented continuing via a minimally invasive approach, and the decision was made to convert to a laparotomy. A 270-degree small bowel volvulus with a 130 cm intussusception was found, located 80 cm from the ligament of Treitz where an endoluminal fatty mass was identified. Segmental resection and primary anastomosis were performed (Fig. 3). No complications were evidenced during the early or late postoperative periods.

The patient was evaluated in the outpatient clinic 15 days after surgery, where the histopathological study reported an intraluminal mass compatible with a lipoma without cellular atypia. At the final postoperative follow-up 6 months after discharge, the patient remained asymptomatic, and definitive discharge was granted.

## Discussion

GL are benign neoplasms of mesenchymal origin composed of mature adipose tissue, with a reported incidence ranging from 0.035% to 4.4%. Although they can locate in any segment of the digestive tract, they show a marked predilection for the colon (65%–75% of cases). They are less frequent in the SB, but their prevalence increases toward the distal ileum. Most of these lesions are small and remain asymptomatic; however, when they exceed 4 cm, they are termed GGL, and 75% of patients develop symptoms such as abdominal pain, obstruction, or bleeding [4, 6, 7].

The incidence of GL does not show a significant gender predilection, distributing equitably between women and men. Nonetheless, some retrospective case series suggest a slight female predominance, with reports indicating up to a 64% prevalence in women. Regarding the age of presentation, although they can be diagnosed at any stage of life, they manifest most frequently between the fifth and seventh decades of life, with a mean age at diagnosis of 67 years. This late onset reinforces the importance of considering GL as a key pathological guide in adults presenting with obstruction or intussusception [4, 6, 8].

Adult II is a rare entity that accounts for only 1% to 5% of intestinal obstruction causes. The presentation of a volvulus associated with an II, as observed in this case, is extremely rare and carries an imminent risk of ischemic necrosis due to vascular compromise of the mesentery. Symptoms are usually non-specific, manifesting as intermittent partial occlusion episodes that complicate the initial clinical diagnosis [6, 9, 10].

Preoperative diagnosis relies primarily on imaging and endoscopic studies. Abdominal CECT is highly specific, showing masses with characteristic fat attenuation (between −40 and −120 Hounsfield units). Endoscopically, classic signs such as the ‘pillow sign’ or the ‘naked fat sign’ after a biopsy have been described, helping confirm the submucosal origin of the lesion. However, in the context of an acute abdomen due to II, CECT is usually the decisive method for planning immediate surgical intervention [8, 11].

The gold standard for treating GL or those causing mechanical complications is segmental surgical resection. Surgery, whether open or laparoscopic, allows not only the resolution of the obstruction and restoration of intestinal transit but also provides a complete histopathological specimen to definitively rule out malignant lesions, such as liposarcoma, which can mimic the appearance of a benign lipoma [1, 4, 12].

## Conclusion

Although histologically benign, GGL pose a critical clinical challenge due to their potential to trigger life-threatening small bowel obstruction and intussusception. Studying these rare presentations is essential to understanding adult surgical morbidity. Prompt surgical resection remains indispensable; it resolves mechanical obstruction, prevents recurrence, and provides crucial histopathological confirmation to rule out malignancy.
